# Bandgap renormalization in single-wall carbon nanotubes

**DOI:** 10.1038/s41598-017-11767-z

**Published:** 2017-09-11

**Authors:** Chunhui Zhu, Yujie Liu, Jieying Xu, Zhonghui Nie, Yao Li, Yongbing Xu, Rong Zhang, Fengqiu Wang

**Affiliations:** School of Electronic Science and Engineering and Collaborative Innovation Center of Advanced Microstructures, Nanjing University, Nanjing, 210093 China

## Abstract

Single-wall carbon nanotubes (SWNTs) have been extensively explored as an ultrafast nonlinear optical material. However, due to the numerous electronic and morphological arrangements, a simple and self-contained physical model that can unambiguously account for the rich photocarrier dynamics in SWNTs is still absent. Here, by performing broadband degenerate and non-degenerate pump-probe experiments on SWNTs of different chiralities and morphologies, we reveal strong evidences for the existence of bandgap renormalization in SWNTs. In particularly, it is found that the broadband transient response of SWNTs can be well explained by the combined effects of Pauli blocking and bandgap renormalization, and the distinct dynamics is further influenced by the different sensitivity of degenerate and non-degenerate measurements to these two concurrent effects. Furthermore, we attribute optical-phonon bath thermalization as an underlying mechanism for the observed bandgap renormalization. Our findings provide new guidelines for interpreting the broadband optical response of carbon nanotubes.

## Introduction

Single-wall carbon nanotubes (SWNTs) exhibit fascinating physical properties that are highly relevant to optoelectronic and photonic applications^1–7^. One prerequisite for engineering SWNT based-optical devices is the understanding of their photophysics on an ultrafast timescale. For instance, tracking ultrafast charge transfer processes between different SWNTs or SWNT-Si heterojunctions can provide important information for designing SWTN-based photovoltaic devices^8, 9^. Furthermore, the ultrafast dynamics of SWNTs offers reliable optical switching mechanism for laser mode-locking^3, 4, 10–12^. Although there are numerous reports on ultrafast carrier dynamics of carbon nanotubes in the literature^13–25^, correlating the experimental results with the underlying physical mechanisms has proved a challenge as it is affected by a number of factors, including SWNT chirality, dielectric medium surrounding the tubes and the experimental configurations for the transient absorption spectroscopy. For example, while ample experimental evidence has confirmed the excitonic nature of optical excitation of SWNTs^26–28^, photo-generation of free carriers has been demonstrated by broadband pump-probe experiments^25^, and the actual role of free carriers in the relaxation processes of SWNTs has not been well understood. In addition, theoretical studies indicate that the electron-hot optical phonon coupling can lead to bandgap renormalization effect in carbon-based materials^29, 30^, however this effect has been largely neglected by previous ultrafast spectroscopy investigations. Another open question concerns the physical origin of photoinduced absorption (PA) signals. By performing non-degenerate pump-probe measurements, Korovyanko *et al*. found a structured PA band in the spectral region between *S*_11_ and *S*_22_ excitonic peaks^16^. Recently, degenerate pump-probe experiments carried out on similar SWNTs show photoinduced bleaching (PB) signatures in the same spectral region^24^. Up until now, it remains a significant challenge to account for the rich photocarrier dynamics of SWNTs using a simple and self-contained physical picture.

Here, to elaborate the broadband photocarrier dynamics of SWNTs and to ensure only intrinsic optical response is probed, we performed broadband (1–2.4 μm) degenerate and non-degenerate pump-probe measurements on SWNTs of different chiralities and morphologies. It is found that the excitonic peaks are always characterized by strong PB signals, while at the red side of the exciton resonances the signals tend to form a PA band. Through spectral and temporal analysis, we conclude that the observed PA signatures originate from a transient bandgap renormalization. Furthermore, our results indicate that the bandgap renormalization is likely to be caused by electron-hot phonon interactions according to Allen, Heine and Cardona’s (AHC’s) theory^29–33^, in contrast to the hot electron−hole plasma origin that has been identified in other low-dimensional systems^34–36^. This work provides an important new framework for interpreting existing results on ultrafast optical response of SWNTs.

## Results

Two SWNT species with different chirality and diameter distributions, as synthesized by the HiPco and the Arc-discharge method, are used to study the ultrafast photocarrier dynamics of SWNTs. We chose to study SWNT ensembles because it is the material form most widely used in emerging optical applications, e.g. saturable absorbers and photodetectors^1–7, 37, 38^. Three SWNT samples, two with polymer matrix and one without, were prepared using a solution processing method (see Methods and Table 1). No aggregates or large bundles were discernible in the SWNT-CMC films^24^. Figure S1 shows the linear absorption spectra for three samples. The excitonic resonance peaks are clearly visible, and can be attribute to *S*_11_ and *S*_22_ transitions^39^.

**Table 1:** The details of three SWNT samples.

Sample	SWNT morphology	Fabrication method	SWNT purity	Diameter range	Matrix	Supplier
HiPco-CMC	Ensemble	HiPco	95%	0.8–1.2 nm	CMC	NanoIntegris Inc.
Arc-CMC	Ensemble	Arc-discharge	90%	1.3–1.6 nm	CMC	Carbon solutions Inc.
Arc-w/o-CMC	Ensemble	Arc-discharge	90%	1.3–1.6 nm	None	Carbon solutions Inc.

To gain a general broadband optical switching signature, we first performed both degenerate and non-degenerate pump-probe experiments on sample HiPco-CMC and Arc-CMC, across 1.0 and 2.4 μm (1.24–0.52 eV). As shown in Figs S2 and 1, such measurements cover the *S*_11_ transition for sample HiPco-CMC, as well as *S*_11_ and *S*_22_ transitions for sample Arc-CMC. Figure 2 shows the differential transmission spectra (Δ*T*/*T*_0_) at time zero for both measurements. While the excitonic peaks are always characterized by strong PB signals regardless of degenerate or non-degenerate configurations as expected from previous literature^25^, the PA signals are found to be more complicated. A generalizable pattern however is discernible, i.e. the PA signatures tend to form a broad band at the low-energy side of the exciton resonances. We then extract the relaxation time constants for each probe wavelength. The relaxation time constants of PB are dependent on probe wavelength and experimental configurations, while the recovery times of PA are featureless and nearly identical, with a time constant ~1.5 ps (Fig. 3). Such a unifying time constant suggests that the PA signals share a common origin.

**Figure 1:**
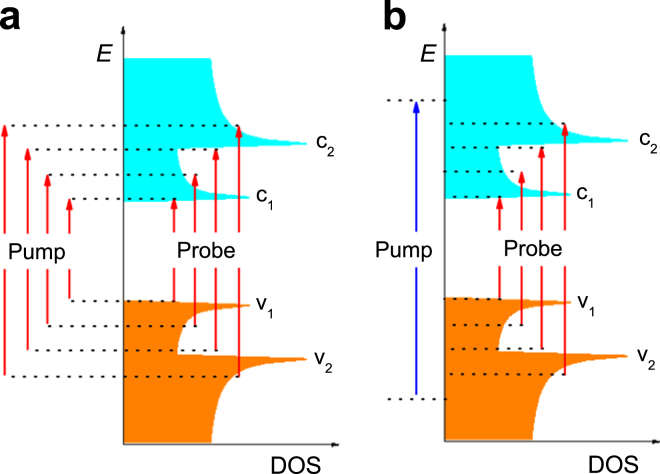
Energy diagram showing the measurements covering *S*_11_ and *S*_22_ for sample Arc-CMC. (**a**) Degenerated configuration. (**b**) Non-degenerated configuration. *c*_n_/*v*_n_ represent *n*-th conduction or valence subbands.

**Figure 2:**
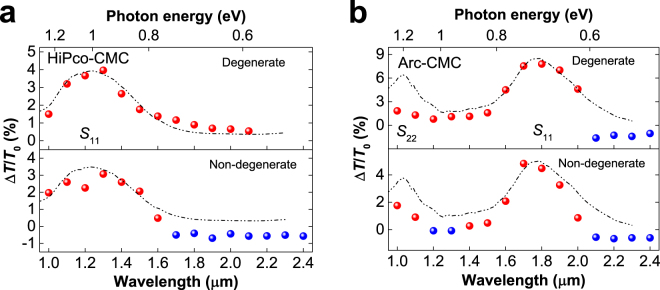
Differential transmission spectra (Δ*T*/*T*_0_) at time zero for sample HiPco-CMC (**a**) and sample Arc-CMC (**b**) with the degenerate (upper panel) and non-degenerate (lower panel) results, pump fluence ~300 μJ/cm^2^. The non-degenerate measurements are performed by using 800 nm pulses excitation. For the sample HiPco-CMC (**a**), the degenerate measurements always exhibit PB signatures (red points) and the PB signal vanishes at a wavelength about 2.2  μm, while the non-degenerate experiments are characterised by the emerging of a PA signal (blue points) at the red-side of *S*_11_, ~1.7 μm. For the sample Arc-CMC (**b**), both measurements show a PA signal at the red-side of *S*_11_, but the non-degenerate experiment also give a PA signal at the red-side of *S*_22_. For the same wavelength range, only PB is observed in the degenerate experiments. The linear absorption of the SWNTs are shown as a visual guide.

**Figure 3:**
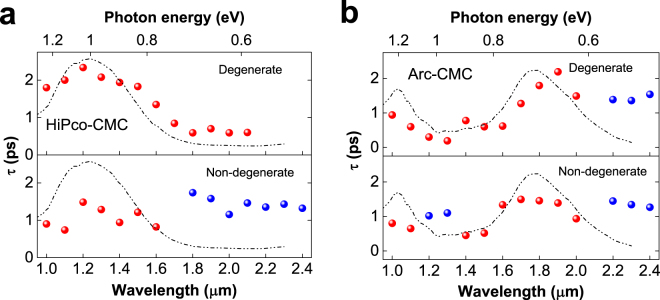
The fitted recovery time constants as a function of probe wavelength for sample HiPco-CMC (**a**) and sample Arc-CMC (**b**) with the degenerate (upper panel) and non-degenerate (lower panel) results. The relaxation time constants of PB (red points) are dependent on probe wavelength and experimental configurations, while the recovery times of PA (blue points) are featureless and nearly identical, with a time constant ~1.5 ps, indicating that the PA signals share a common origin.

## Discussion

Probe wavelength dependent photo-bleaching and photo-absorption signatures were revealed by previous pump-probe investigations and described by different mechanisms, such as intersubband transitions^16^, multiple-exciton formation^40–42^, charge induced Stark effect^43–45^ and global redshift of the π-plasmon resonance^15^. However, none of these mechanisms can explain our data very well. The broad PA signature rule out intersubband transitions and multiple-exciton formation, as both should correspond to a structured PA peak^16, 40^. Charge induced Stark effect is generated by long-lived charge carriers^43–45^, however no long-time PA signals were observed in our measurements. In addition, by performing a 400 nm pump-probe measurement (Fig. S3), we identify a PB-PA transition at ~2 µm wavelength, clearly within the *S*_11_ band of sample Arc-CMC. It challenges the non-resonance framework of global redshift of the π-plasmon resonance^15^. A more generic mechanism for the observed features needs to be elaborated.

It is known that SWNTs present very strong many-body interactions due to the quantum confinement effect^46, 47^. Hence, we argue that the many-body effects should be taken into account for comprehending the optical switching properties of SWNTs. Additional photocarriers can alter the screening of the Coulomb potential, resulting in both a reduction of exciton binding energy and electronic bandgap shrinkage^34–36, 48^. However, the reduction of exciton binding energy and electronic bandgap shrinkage usually are of similar size^48^, thus no shift of the absorption spectrum is observed until the excited carrier density approaching the Mott transition density, where the exciton resonance no longer exists^35^. However, for our experimental pump fluence, the PB signals are found following the exciton resonance (as shown in Figs 2 and S3). Hence, it is safe to conclude that there is no Mott transition in out measurements, and carrier effect should not be responsible for the observed PA signals.

**Figure 4:**
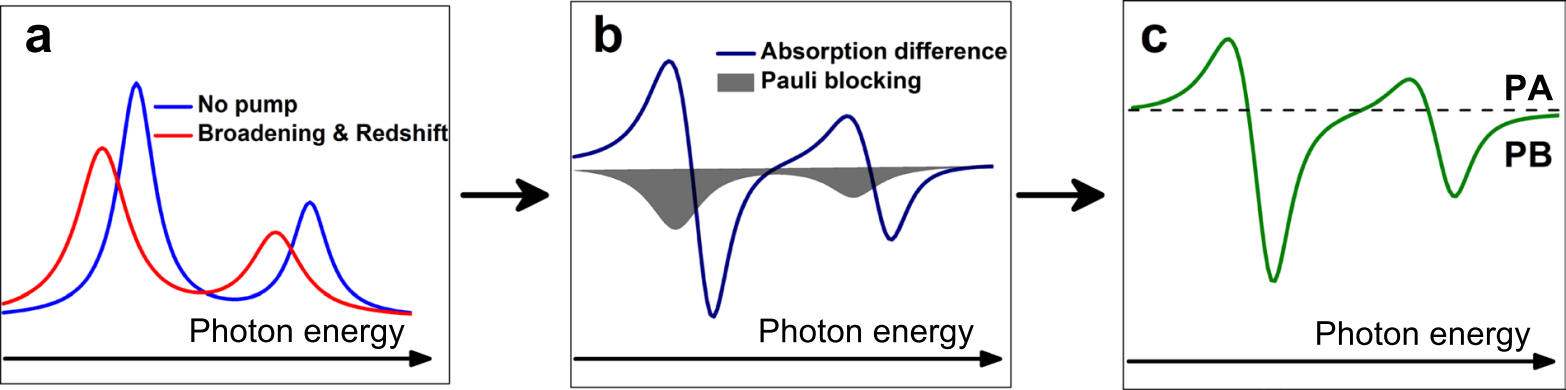
Illustration of the combined effects of Pauli blocking and bandgap renormalization. (**a**) Ground-state absorption spectrum and excited-state absorption spectrum based on bandgap renormalization effects (line broadening and redshift). (**b**) Bandgap renormalization induced absorption difference versus Pauli blocking. (**c**) Transient absorption spectrum.

Another many-body effect stem from carrier-phonon coupling^49–52^. The hot-carrier of SWNTs cool down by emitting optical phonons that lead to very fast phonon bath thermalization with a timescale <100 fs^53–55^. As we noticed before, the quasiparticle band structures of materials can be renormalized by increasing optical-phonon temperature^29–33^. For example, bandgap redshift by ~60 meV and broadening parameters differing by ~30 meV was obtained for diamond^29^. Figure 4 depicts the idea, which illustrate transient absorption spectra based on the bandgap renormalization (redshift and linewidth broadening) as well as Pauli blocking. Good qualitative agreement with the key features of our experimental pump-probe signal was obtained. Therefore, we conclude that the observed optical switching features maybe attribute to a bandgap renormalization, caused by the electron-hot optical phonon interactions. This assignment is also supported by the following arguments. First, such an interpretation agrees with the previous Raman investigation^49, 51^. By increasing the laser power, Fantini *et al*. found that *S*_11_ peaks are broadened and red-shifted, and *S*_22_ energies also present an average 70 meV red-shift, although it is red-shifted for the tubes with (2n + m) mod 3 = 1 and blue-shifted for the tubes with (2n + m) mod 3 = 2^49^. Second, the relaxation time constants of PA signals coincide with the optical phonon lifetimes about 1–2 ps^55–58^.

**Figure 5:**
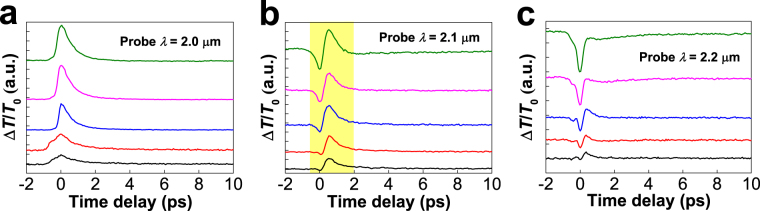
The pump fluence-dependent non-degenerate results for sample Arc-w/o-CMC with three selected probe wavelengths (**a**) 2.0 μm, (**b**) 2.1 μm, and (**c**) 2.2 μm. Form bottom to top, the pump fluences are 0.13, 0.3, 0.62, 1.05 and 1.7 mJ/cm^2^, respectively.

It is worth pointing out that the distinct signatures obtained from degenerate and non-degenerate measurements (e.g. Fig. 2b, refs 16 and 24) may be as a result of different sensitivity to the Pauli blocking and bandgap renormalization effects. The degenerate measurements more closely represent the behaviour of a two-level model depicting saturable absorption at the probe wavelength. However, the optical response from the non-degenerate experiments actually probed a combined effect of Pauli blocking and bandgap renormalization, since they involve both excited states and carrier relaxation pathways^24^. To provide further insights into the competition mechanism between these two effects, we performed pump fluence-dependent non-degenerate measurements on sample Arc-w/o-CMC. As shown in Figs 5 and S4, at a critical wavelength (e.g. ~2.1 µm), we observe a non-trivial delay time-dependent PA-PB transition, i.e. the signal is negative (PA) immediately after pump excitation and flips to positive (PB) within about 1 ps. At slight shorter wavelength (~2.0 µm), only PB signal is detected, whereas at slight longer wavelength (2.2 µm), PA can completely override the weak PB signal. The change of transient dynamics as shown in Fig. 5 provides direct evidence for the existence of competition between Pauli blocking and bandgap renormalization effects. In Fig. S5, we summarize the peak value of PA and PB extracted from the transient dynamics for a probe wavelength at 2.1 µm (Fig. 5b). The peak value of PA signal is seen to linearly increase with the increasing of pump fluence, while the PB signal readily saturates at a moderate pump fluence ~600 µJ/cm^2^. Such features indicate that the comparative weight between Pauli blocking and bandgap renormalization is highly dependent on pump fluences, which should be used as an important control parameter in interpreting the ultrafast dynamics of SWNTs. It should be pointed out that although we have demonstrated the competition between Pauli blocking and bandgap renormalization effects, the hot-optical phonon bath build-up signature is not directly observed in our measurements. This is probably due to the limited temporal resolution of our current setup (~300 fs).

In summary, we have performed broadband (1–2.4 µm) pump-probe measurements on SWNTs of different chiralities and morphologies with both degenerate and non-degenerate configurations. Two common optical switching features are obtained, where photo-bleaching is found at excitonic transitions and a photoinduced absorption band tends to form at the red-side of exciton resonance. More importantly, these results have been well accounted for by considering bandgap renormalization effects, caused by the electron-hot optical phonon coupling. Our work provides a simple physical model to interpret the broadband transient optical response of carbon nanotubes.

## Methods

### SWNT sample preparation

Raw SWNT powers were initially dispersed in N-methyl-2-pyrrolidone (NMP) by an ultrasonic degradation. Then, the dispersions were centrifuged at 5000 rpm for 1 hour to remove large SWNT bundles. Sample Arc-w/o-CMC was subsequently fabricated on silica substrate by dip coating followed by an overnight bake at 60 °C, while for samples Arc-CMC and HiPco-CMC, the supernatants were mixed with Carboxymethyl cellulose (CMC) and the film samples were finally synthesized by gradually evaporating the solvent for several days.

### Pump-probe measurement

An 800 nm, 1 kHz Ti: Sapphire amplifier (Libra, Coherent Inc.) was used as laser source. The 400 nm pulses were obtained by frequency doubling in a *β*-Barium Borate (BBO) nonlinear crystal, and the infrared (1–2.4 μm) pulses were generated by feed a portion of 800 nm pulses into an optical parametric amplifier system (OPA-SOLO, Coherent Inc.). Both degenerate and non-degenerate pump–probe setup was based on a transmission geometry. The pump pulses passed through a 334 Hz chopper so that we could record pump-induced differential transmission changes Δ*T*/*T*_0_ by the lock-in amplifying technique. For all the measurements, the used pump fluence was about 300 μJ/cm^2^ unless specified otherwise. The time resolution of our setup was about 300 fs, and the relaxation time constants were extracted by a mono-exponential decay function fitting. All of the measurements were performed at room temperature and ambient environment.

### Data availability

All important data supporting the findings of this study are included in this published article (and its Supplementary Information files). Further data sets are available from the corresponding author on reasonable request.

## Electronic supplementary material

Supplementary information
