# Supplementary material for: Bandgap renormalization in single-wall carbon nanotubes
Source: Sci Rep. 2017 Sep 11;7:11221. doi: 10.1038/s41598-017-11767-z (PMC5593862; doi:10.1038/s41598-017-11767-z)
Supplement: Supplementary file 1 — Supplementary information [file 41598_2017_11767_MOESM1_ESM.doc]

**SUPPLEMENTARY INFORMATION**

**Bandgap renormalization in single-wall carbon nanotubes**

Chunhui Zhu, Yujie Liu, Jieying Xu, Zhonghui Nie, Yao Li, Yongbing Xu,

Rong Zhang, Fengqiu Wang*

School of Electronic Science and Engineering and Collaborative Innovation Center of Advanced Microstructures, Nanjing University, Nanjing 210093, China.

E-mail: fwang@nju.edu.cn

**Figure S1 |** The linear absorption spectra for three samples.

**Figure S2 |** Energy diagram showing the measurements covering *S*_11_ for sample HiPco-CMC. (**a**) Degenerated configuration. (**b**) Non-degenerated configuration. *c*_n_/*v*_n_ represent *n*-th conduction subbands/valence subbands.

**Figure S3 |** Δ*T*/*T*_0_ at time zero as function of wavelengths for sample Arc-CMC with 400 nm pump-infrared probe.

**Figure S4 |** Fluence-dependent non-degenerate pump-probe results for sample Arc-w/o-CMC with six selected probe wavelengths. (**a**) 1.6 μm, (**b**) 1.7 μm, (**c**) 1.8 μm, (**d**) 1 .9 μm, (**e**) 2.3 μm and (**f**) 2.4 μm. Form bottom to top, the pump fluences are 0.13, 0.3, 0.62, 1.05 and 1.7 mJ/cm^2^, respectively.

**Figure S5 |** The peak value of PA and PB obtained from non-degenerate measurements for a probe wavelength of 2.1 µm.
